# Synthesis, characterization and in vitro antimicrobial activity of novel fused pyrazolo[3,4-*c*]pyridazine, pyrazolo[3,4-*d*]pyrimidine, thieno[3,2-*c*]pyrazole and pyrazolo[3′,4′:4,5]thieno[2,3-*d*]pyrimidine derivatives

**DOI:** 10.1186/s13065-017-0339-4

**Published:** 2017-11-02

**Authors:** Mohamed A. M. Abdel Reheim, Safaa M. Baker

**Affiliations:** Department of Chemistry, Faculty of Science, Arish University, Arish, 45511 Egypt

**Keywords:** Substituted pyrazolone, Pyrimidine derivatives, Antimicrobial activity

## Abstract

**Background:**

Some novel substituted pyrazolone, pyrazolo[3,4-c]pyridazine, pyrazolo[3,4-d]pyrimidine, pyrazolo[3,4-*d*]thiazolo[3,2-*a*]pyrimidinone, thieno[3,2-*c*]pyrazole and pyrazolo[3′,4′:4,5]thieno[2,3-*d*]pyrimidine derivatives have been reported to possess various pharmacological activities like antimicrobial, antitumor and anti-inflammatory.

**Results:**

A novel series of azoles and azines were designed and prepared via reaction of 1,3-diphenyl-1*H*-pyrazol-5(4*H*)-one with some electrophilic and nucleophilic reagents. The structures of target compounds were confirmed by elemental analyses and spectral data.

**Conclusions:**

The antimicrobial activity of the target synthesized compounds were tested against various microorganisms such as *Escherichia coli*; *Bacillus megaterium*; *Bacillus subtilis* (Bacterial species), *Fusarium proliferatum*; *Trichoderma harzianum*; *Aspergillus niger* (fungal species) by the disc diffusion method. In general, the novel synthesized compounds showed a good antimicrobial activity against the previously mentioned microorganisms.

## Background

The compounds containing nitrogen are important category of heterocyclic compounds, which play a significant roles in modern pesticide industry (85% of pesticides with high activity and low toxicity contain nitrogen heterocyclic compound) [1]. Pyrazoles are important moieties as building blocks for many heterocyclic products and act as abinucleophile [2] with abroad spectrum of remarkable biological activities. Many derivatives containing pyrazole nucleus have been commercialized as herbicides, insecticides and fungicides for plant protection [3]. Heterocycles containing a pyrazole or pyrazolone nucleus have been reported to show abroad spectrum of biological activity including antimicrobial [4], anti-cyclooxygenase [5], anti-convulsant [6], antitubercular [7], antitumor [8], anti-inflammatory [9], analgesic [10], antidiabetic [11], antipshycotic [12–14]. In last few years, we have been involved in a program aimed at developing new efficient synthetic approaches for the synthesis of heterocyclic compounds of biological interest [15–17]. Since most of the pyrazole derivatives show anti-microbial activity, the synthesized compounds are also expected to show antimicrobial activity. Hence, our plan is to synthesize some substituted pyrazole derivatives and subsequently screen for their antimicrobial activity.

## Results and discussion

### Chemistry

The starting material 4-acetyl-1, 3-diphenyl-1*H*-pyrazol-5(4*H*)-one **2** was synthesized from acylation of pyrazolone **1** [18] with acetyl chloride in acetic anhydride and sodium acetate under reflux in good yield [19, 20].

**Scheme 1:**
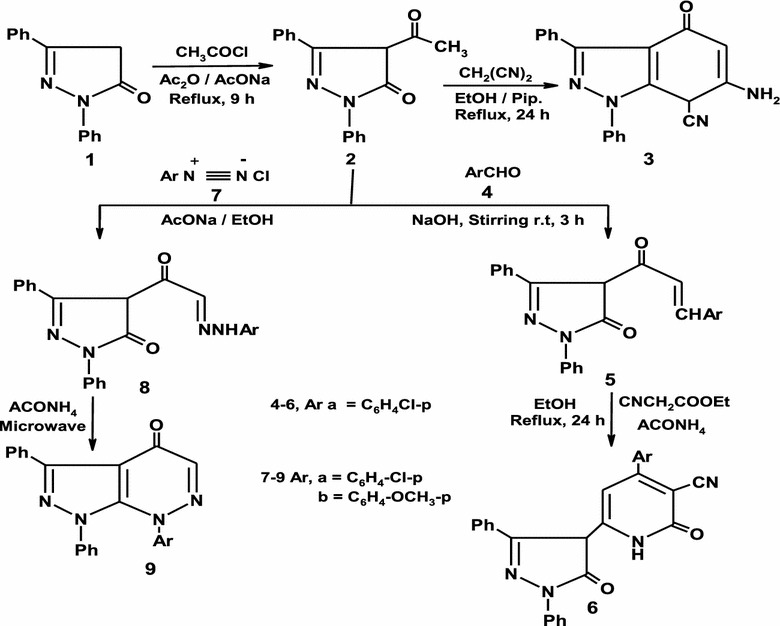
Synthesis of pyrazoles 2–9

Pyrazol-5-one derivative **2** was exploited as a key intermediate for the synthesis of hitherto unknown fused pyrazole. Thus cyclocondensation of **2** with active methylene reagent such as malononitrile in ethanol under reflux in the presence catalytic amount of piperidine afforded indazole derivative **3** on the basis of analytical and spectral data (Scheme 1). The formation of **3** from the reaction of **2** with malononitrile is believed to be formed via initial condensation of malononitrile with the ring carbonyl and subsequent elimination of water followed by addition of methyl group on the triple bond system of cyano group. Also, compound **2** condensed with aryl aldehyde **4a** in ethanol containing 10% sodium hydroxide to afford the condensation product **5** based on its elemental and spectral data (Scheme 1) [21]. Cyclization of **5** with ethyl cyanoacetate in ethanol in the presence of ammonium acetate at reflux temperature led to the formation of dihydropyridine derivative **6** (Scheme 1) [22–25]. The reactivity of methyl group in pyrazolone **2** toward aryl diazonium salts was also investigated aiming at preparation of new pyridazine derivatives. Thus, when **2** coupled with aryl diazoniuum salt **7a** in ethanol in the presence of sodium acetate yielded hydrazone **8a** on the basis on its spectral data. The ^1^H-NMR spectrum of compound **8a** recorded in DMSO-*d*_*6*_ revealed a signal at δ = 12.00 ppm which could be attributed to hydrazone NH group. Similarly, pyrazolone **2** was coupled readily with aryl diazonium salts **7b** in the same reaction conditions to give **8b** as demonstrated in (Scheme 1). Compounds **8a–b** could be cyclized to the corresponding pyrazolo[3,4-*c*]pyridazin-4(7H)-one **9a–b** upon fusion in domestic microwave oven in the presence of ammonium acetate (Scheme 1) [26, 27].

**Scheme 2:**
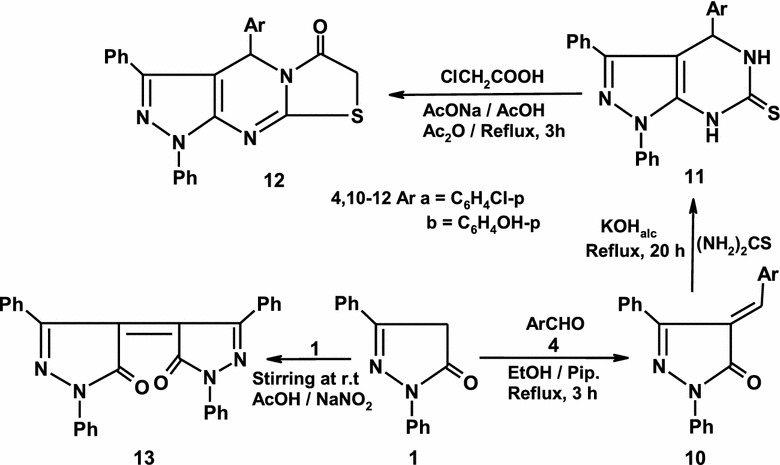
Synthessis of pyrazoles 10–13

The foregoing results prompt us to investigate the synthetic potentiality of pyrazolone **1** toward a variety of electrophilic reagents. Thus, when pyrazolone **1** was allowed to react with aryl aldehydes **4a–b** to give arylidines **10a–b**. The pyrazolopyrimidines **11a–b** were obtained by cyclization of pyrazolones **10a–b** with thiourea in refluxing ethanol containing 10% potassium hydroxide (Scheme 2). The formation of pyrazolopyrimidinethione **11** is believed to be formed via initial condensation of thiourea with the carbonyl group of **10** and subsequent elimination of water followed by addition NH_2_ of thiourea on the double bond system of **10** [21, 28–31]. Pyrazolopyrimidinethiones **11a–b** was used as building blocks for the synthesis of condensed heterocycles. Thus, when pyrazolopyrimidinethione **11a** is allowed to react with chloroacetic acid in refluxing acetic acid in the presence of sodium acetate furnished pyrazolo[3,4-*d*]thiazolo[3,2-*a*]pyrimidine derivative **12a** in a quantitative yield (Scheme 2). Similarly, pyrazolopyrimidinethione **11b** reacted with chloroacetic acid in the same reaction condition to give pyrazolo[3,4-*d*]thiazolo[3,2-*a*]pyrimidine derivative **12b** (Scheme 2) [32–34]. Diphenylpyrazolone **1** was oxidized by exposing it to air to give 4-(5-oxo-1, 3-diphenyl-1*H*-pyrazol-4(5*H*)-ylidene)-1,3-diphenyl-1*H*-pyrazol-5-one **13** (scheme 2) [35].

**Scheme 3:**
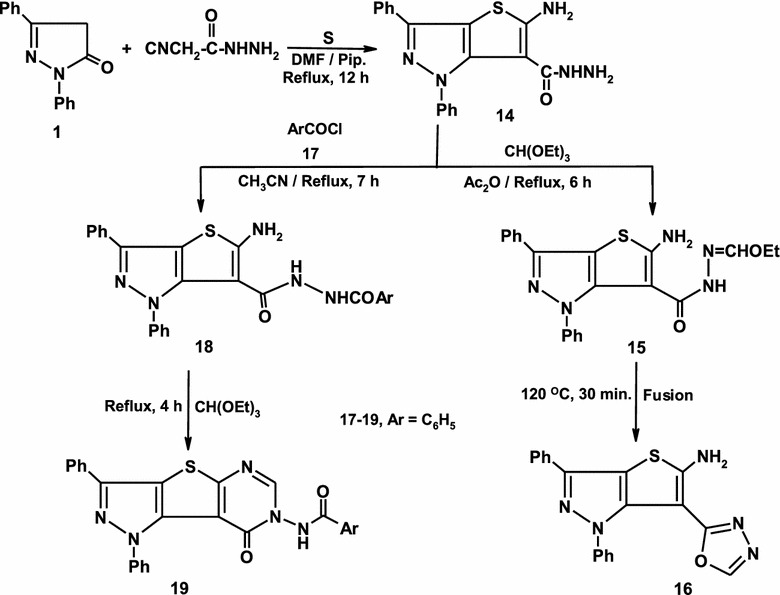
Synthesis of pyrazoles 14–19

As an extension to *Gewald* synthesis of thiophene and fused thiophene, a mixture of diphenyl pyrazolone **1**, cyanoacetic acid hydrazide and elemental sulfur in DMF containing a catalytic amount of piperidine is refluxed to yield 5-amino-1,3-diphenyl-1*H*-thieno[3,2-*c*]pyrazole-6-carbohydrazide **14** based on its elemental and spectral data (Scheme 3) [36].

Hydrazide **14** is used as a key precursor for many chemical transformations to synthesize a variety of important heterocycles. Thus, when compound **14** was allowed to react with triethylorthoformate in refluxing acetic anhydride afforded 5-amino-1,3-diphenyl-1H-thieno[3,2-c]pyrazole-6-(N-ethoxymethylene-carbohydrazide) **15** (Scheme 3). Fusion of **15** afforded 6-(1,3,4-oxadiazol-2-yl)-1,3-diphenyl-1*H*-thieno[3,2-*c*]pyrazol-5-amine **16**. Establishing structure of **16** was based on its elemental and spectral data. For example the infrared spectrum of thienopyrazole **16** revealed the absence of carbonyl group. The ^1^H-NMR of the same product revealed absence of signals of ethyl fragment. The mass spectrum showed a very intense molecular ion peak at 361 (M^+^+2) and a number of fragments support the proposed structure [37]. Treatment of **14** with benzoyl chloride **17** afforded 5-amino-*N*′-benzoyl-1,3-diphenyl-1*H*-thieno[3,2-*c*]pyrazole-6-carbohydrazide **18** on the basis of its elemental analysis and spectral data. Moreover, *the* reaction of **18** with triethylorthoformate at reflux temperature afforded the fused pyrimidine derivative **19** (Scheme 3) [38].

**Scheme 4:**
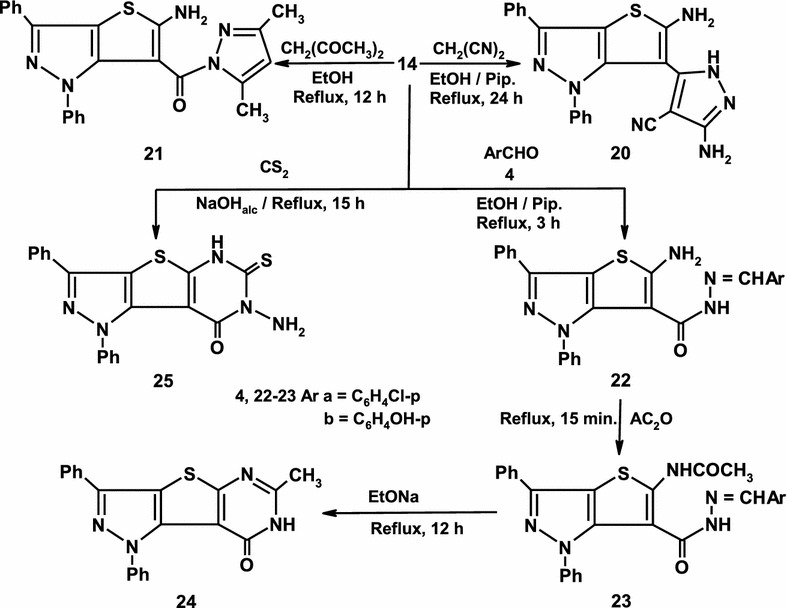
Synthesis of pyrazoles 20–24

The behavior of thienopyrazole **14** toward active methylene reagents was also investigated. Thus, thienopyrazole **14** was reacted with malononitrile in refluxing ethanol containing catalytic amount of piperidine to yield 3-amino-5-(5-amino-1,3-diphenyl-1*H*-thieno[3,2-*c*]pyrazol-6-yl)-1*H*-pyrazole-4-carbonitrile **20** (Scheme 4). The formation of **20** is believed to be formed via condensation of malononitrile with carbonyl group of **14** followed by addition of amino group on the cyano group of malononitrile and subsequent cyclization to give **20**. Also thienopyrazole **14** reacted with acetylacetone in refluxing ethanol to afford 5-amino-1,3-diphenyl-1*H*-thieno[3,2-c]pyrazol-6-yl)(3,5-dimethyl-1*H*-pyrazol-1-yl)methanone **21** based on its elemental and spectral data (Scheme 4). Furthermore, treatment of compound **14** with aryl aldehydes **4a–b** yielded arylmethylene hydrazide derivatives **22a–b** in quantitative yields [39]. Acylation of **22a–b** using acetic anhydride under reflux afforded **23a–b** which undergoes cyclization upon refluxing in sodium ethoxide to afford the pyrazolo[3′,4′:4,5]thieno[2,3-d]pyrimidinone derivative **24** (Scheme 4) [37]. Finally, compound **14** was treated with carbon disulphide in refluxing ethanol/sodium hydroxide solution to afford the promising compound 7-amino-1,3-diphenyl-6-thioxo-1,5,6,7-tetrahydro-8*H*-pyrazolo[3′,4′:4,5]thieno [2,3-*d*]pyrimidin-8-one **25** (Scheme 4). Establishing structure **25** was based on its elemental and spectral data.

### Antimicrobial activity

The newly synthesized compounds and their derivatives have been screened for antibacterial activity against some gram negative bacteria (*Escherichia coli*) and some gram positive bacteria (*Bacillus megaterium* and *Bacillus subtilis*), and antifungal activity against *Fusarium proliferatum*, *Trichoderma harzianum* and *Aspergillus niger*, by the cup-plate method and agar diffusion disc method for determining MIC (minimum inhibitory concentration), ampicillin and colitrimazole were used as standards for comparison of antibacterial and antifungal activity, respectively.

The anti-bacterial activity of the synthesized compounds was tested against bacterial species (*E. coli*; *B. megaterium*; *B. subtilis*) and the antifungal activity was tested also against fungal species (*F. proliferatum*; *T. harzianum*; *A. niger*). Each compound was dissolved in DMF, About 100 mL of each compound will be pipetted and poured into the cups existed in nutrient agar plates containing medium which consisted of: peptic digest of animal tissue 5.00, sodium chloride 5.00, Beef extract 1.50, Yeast extract 1.50, Agar 15.00 all in gm/L, final pH at 25 °C; 7.4 ± 0.2) or Czapek’s agar plates for fungi (sucrose 30.00, sodium nitrate 2.00, dipotassium phosphate 1.00, magnesium sulphate 0.50, potassium chloride 0.50, ferrous sulphate 0.01, Agar 15.00, all in gm/L, final pH at 25 °C; 7.3 ± 0.2), seeded with *E. coli*, *B. megaterium* and *B. subtilis*, *F. proliferatum, T. harzianum* and *A. niger*, respectively.

For determining minimum inhibitory concentration (MIC), serial dilutions of tested compounds (μg/mL) as well as reference antibiotics were prepared using 10% DMF solution, paper discs of Whatman filter paper were prepared with standard size (8 mm), were cut and sterilized in an autoclave. The paper discs soaked in the desired compound solution were placed aseptically in the petri dishes containing agar media and microbial species. The petri dishes were incubated at 36–37 °C and the inhibition zones were recorded after 24 h of incubation in case of bacteria and after 5–7 days in case of fungi. Each treatment was replicated three times [40, 41]. The antibacterial activity of a common standard antibiotic ampicillin and antifungal Clotrimazole was also recorded using the same procedure as above at the same concentration and solvents. The % activity index for the compound was calculated by the following formula.

$$\% {\text{ Activity index }} = \frac{{{\text{Zone of inhibition by test compound }}\left( {\text{diameter}} \right)}}{{{\text{Zone of inhibition by standard }}\left( {\text{diameter}} \right) \times \; 100}}$$

Our results showed that most of checked compounds were active against most of micro-organisms used, while the discs which containing DMF solution (10%) alone were not exhibited any effect on the growing micro-organisms (no inhibition zone around the discs). The results of antimicrobial and antifungal activity and its MIC are illustrated in Tables 1, 2. We found that compounds; **3**, **13**, **2**, **12a** and **20** showed promising broad spectrum antibacterial activities against *E. coli*. Compounds **14**, **12b**, **15**, **2** and **24** showed maximum antimicrobial activity against *B. megaterium*, *B. subtilis*, *F. proliferatum*, *T. harzianum* and *A. niger*, respectively. Compounds; **9b**, **8b**, **6**, **22a**, **5a**, **11b**, **18** and **16** demonstrated moderate antimicrobial activity against gram positive, gram negative bacteria and fungi. On the other hand, **10a**, **10b**, **11a**, **23a**, **25** and **23b** exhibited low antibacterial activity and moderate to low antifungal activity, whereas **25** and **23b** showed high antibacterial activity against only *B. subtilis*. From Table 2, we observed that compounds; **13**, **6**, **3** and **14** showed the minimum inhibitory concentrations (MIC) for most tested bacteria and fungi, while compounds; **9b**, **8b**, **22a**, **5a**, **11b**, **18** and **19** exhibited high concentrations of MIC as compared with standard antimicrobial agents used.

**Table 1:** Antibacterial and antifungal activities of synthesized compound

Compounds	Bacterial species						Fungal species					
	*Escherichia coli*		*Bacillus megaterium*		*Bacillus subtilis*		*Fusarium proliferatum*		*Trichoderma harzianum*		*Aspergillus niger*	
	Inhibition zone diameter (mm)	% activity index	Inhibition zone diameter (mm)	% activity index	Inhibition zone diameter (mm)	% activity index	Inhibition zone diameter (mm)	% activity index	Inhibition zone diameter (mm)	% activity index	Inhibition zone diameter (mm)	% activity index
10a	10	43.48	10	43.48	15	65.22	10	45.45	12	54.55	15	68.18
10b	10	43.48	NA	0.00	15	65.22	12	54.55	12	54.55	10	45.45
11a	10	43.48	10	43.48	NA	0.00	12	54.55	15	68.18	15	68.18
11b	12	52.17	NA	0.00	NA	0.00	NA	0.00	10	45.45	12	54.55
2	15	65.22	12	52.17	NA	0.00	12	54.55	15	68.18	12	54.55
12a	15	65.22	10	43.48	20	86.96	12	54.55	12	54.55	10	45.45
12b	10	43.48	NA	0.00	20	86.96	10	45.45	12	54.55	NA	0.00
8b	10	43.48	10	43.48	20	86.96	12	54.55	10	45.45	15	68.18
3	20	86.96	12	52.17	20	86.96	15	68.18	10	45.45	12	54.55
5a	NA	0.00	NA	0.00	12	52.17	15	68.18	NA	0.00	10	45.45
6	NA	0.00	12	52.17	12	52.17	15	68.18	NA	0.00	NA	0.00
9b	NA	0.00	10	43.48	20	86.96	10	45.45	12	54.55	NA	0.00
13	20	86.96	12	52.17	20	86.96	12	54.55	15	68.18	12	54.55
18	10	43.48	10	43.48	20	86.96	20	90.91	12	54.55	15	68.18
22a	12	52.17	NA	0.00	12	52.17	15	68.18	NA	0.00	10	45.45
20	15	65.22	10	43.48	15	65.22	20	90.91	10	45.45	15	68.18
23a	10	43.48	12	52.17	15	65.22	15	68.18	NA	0.00	12	54.55
23b	12	52.17	12	52.17	20	86.96	12	54.55	10	45.45	0	0.00
25	10	43.48	NA	0.00	20	86.96	10	45.45	10	45.45	0	0.00
24	12	52.17	10	43.48	20	86.96	12	54.55	NA	0.00	15	68.18
15	12	52.17	10	43.48	20	86.96	20	90.91	12	54.55	15	68.18
21	12	52.17	NA	0.00	12	52.17	12	54.55	10	45.45	12	54.55
16	10	43.48	10	43.48	15	65.22	12	54.55	NA	0.00	12	54.55
14	12	52.17	15	65.22	20	86.96	NA	0.00	0	0.00	12	54.55
19	NA	0.00	NA	0.00	15	65.22	15	68.18	10	45.45	0	0.00
Ampicillin (antibacterial standard)	23	100.0	23	100.00	23	100.00	–	–	–	–	–	–
Colitrimazole (antifungal standard)	–	–	–	–	–	–	22	100.0	22	100.00	22	100.00

**Table 2:** Minimum inhibitory concentrations (MIC) for tested compounds

Compounds	Minimum inhibitory concentration (MIC) of the synthesized compounds (µg/mL)					
	Bacterial species			Fungal species		
	*Escherichia coli*	*Bacillus subtilis*	*Bacillus megaterium*	*Fusarium proliferatum*	*Trichoderma harzianum*	*Aspergillus niger*
10a	NA	NA	5.10	10.20	25.51	5.10
10b	NA	NA	1.71	21.43	21.43	NA
11a	NA	NA	23.45	46.90	23.45	23.45
11b	14.84	NA	14.84	14.84	29.67	29.67
2	33.47	33.47	33.47	33.47	33.47	5.36
12a	36.22	36.22	5.80	36.22	72.45	NA
12b	29.18	NA	14.59	NA	29.18	NA
8b	87.76	29.20	7.02	43.88	87.76	43.88
3	NA	87.75	NA	5.71	35.71	35.71
5a	NA	NA	NA	35.49	NA	NA
6	NA	71.43	35.71	35.71	NA	NA
9b	54.69	54.69	4.38	4.38	54.69	54.69
13	4.08	10.00	4.08	NA	8.16	8.16
18	58.16	NA	29.08	29.08	29.08	4.65
22a	30.61	NA	NA	30.61	NA	30.61
20	43.47	86.94	43.47	86.94	NA	86.94
23a	NA	83.67	41.84	41.84	NA	6.69
23b	28.78	57.55	4.60	28.78	57.55	4.60
25	66.33	132.65	66.33	132.65	NA	10.61
24	77.14	77.14	6.17	NA	NA	6.17
15	37.96	NA	18.98	NA	37.96	18.98
21	31.84	NA	15.92	NA	NA	15.92
16	47.96	NA	23.98	NA	NA	3.84
14	21.22	21.22	3.40	42.45	42.45	21.22
19	NA	90.20	45.10	7.22	NA	7.22

*NA* no activity

## Experimental section

### Chemistry

The melting points, the elemental analysis and the spectral data were recorded as reported in references [19].

Synthesis of 4-acetyl-1,3-diphenyl-1*H*-pyrazol-5(4*H*)-one **(2)**. A mixture of pyrazolone **1** (0.01 mol) and acetyl chloride (0.01 mol) in acetic anhydride (10 mL) and sodium acetate (2 gm) was heated under reflux for 9 h. The reaction mixture was allowed to cool and poured into crushed ice then acidified with HCl. The separated solid was filtered, washed with water and crystallized from ethanol to give white crystals; yield (88%); m.p. 111–113 °C. IR (KBr, cm^−1^) ν_max_ = 3062 (CH-arom), 2956 (CH-aliph), 1706, 1690 (2CO) cm^−1^. ^1^H-NMR (300 MHz, DMSO-*d*_*6*_) δ (ppm): 1.91 (s, 3H, CH_3_), 2.32 (s, 1H, CH-pyrazole), 7.37–8.14 (m, 10H, aromatic H). ^13^C-NMR (100 MHz, DMSO-*d*_*6*_) δ (ppm): 27.0, 58.1, 121.6, 121.6, 125.8, 126.1, 126.1, 127.3, 127.3, 127.9, 127.9, 128.8, 135.0, 137.2, 151.3, 161.9, 200. MS (EIMS) *m/z*: 278 (M^+^, 1), 276 (18), 268 (22), 236 (63), 161 (29), 134 (23), 128 (84), 127 (11), 103 (60), 91 (65), 77 (100), 51 (21). Anal. Calcd. for C_17_H_14_N_2_O_2_ (278): C, 73.37; H, 5.07; N, 10.07. Found: C, 73.44; H, 5.12; N, 10.19%.

Synthesis of 6-amino-4-oxo-1,3-diphenyl-4,7-dihydro-1*H*-indazole-7-carbonitrile **(3)**. A mixture of **2** (0.01 mol), malononitrile (0.01 mol) in ethanol (30 mL) containing catalytic amount of piperidine was heated under reflux for 24 h. The reaction mixture was allowed to cool and poured into crushed ice then acidified with HCl. The separated solid was filtered, washed with water and crystallized from ethanol to give brawn crystals; yield (80%); m.p. 170–172 °C. IR (KBr, cm^−1^) ν_max_ = 3447, 3400 (NH_2_), 3058 (CH-arom), 2952 (CH-aliph), 2192 (CN), 1700 (CO) cm^−1^. ^1^H-NMR (400 MHz, DMSO-*d*_*6*_) δ (ppm): 3.63 (s, 1H, CH), 6.02 (s, 1H, = CH), 7.25–7.92 (m, 10H, aromatic H), 11.81 (s, 2H, NH_2_). ^13^C-NMR (100 MHz, DMSO-*d*_*6*_) δ (ppm): 33.1, 108.4, 109.2, 113.8, 123.8, 123.8, 124.2, 125.5, 125.5, 127.6, 128, 128, 128.3, 128.3, 131, 139.7, 141.1, 150.8, 158.5, 180.6. MS (EIMS) *m/z*: 327 (M^+^+1, 0.2), 236 (40), 194 (5), 131 (4), 103 (61), 91 (53), 77 (100), 64 (27), 51 (32). Anal. Calcd. for C_20_H_14_N_4_O (326): C, 73.61; H, 4.32; N, 17.17. Found: C, 73.63; H, 4.34; N, 17.19%.

Synthesis of 4-(3-(4-chlorophenyl)acryloyl)-1,3-diphenyl-1*H*-pyrazol-5(4*H*)-one **(5)**. A mixture of **2** (0.01 mol), 4-chlorobenzaldehyde **4a** (0.01 mol) and 10% aqueous sodium hydroxide (10 mL) in ethanol (50 mL) was stirred at room temperature for about 3 h. The reaction mixture poured into crushed ice then acidified with HCl. The resulting solid was filtered off, washed with water, dried and crystallized from ethanol to give pale yellow crystals; yield (86%); m.p. 170–172 °C. IR (KBr, cm^−1^) ν_max_ = 3060 (CH-arom), 2951 (CH-aliph), 1712, 1692 (2CO) cm^−1^. ^1^H-NMR (300 MHz, DMSO-*d*_*6*_) δ (ppm): 3.34 (s, 1H, CH-pyrazole), 5.24 (d, 1H, = CH), 6.01 (d, 1H, = CH), 7.20–8.54 (m, 14H, aromatic H). ^13^C-NMR (100 MHz, DMSO-*d*_*6*_) δ (ppm): 53.6, 123.0, 123.0, 125.3, 127.7, 127.7, 127.8, 128.0, 128.0, 128.0, 128.0, 128.6, 128.6, 129.1, 129.1, 130.2, 130.2, 130.8, 135.0, 137.7, 140.5, 152.6, 166.3, 198.6. MS (EIMS) *m/z*: 400 (M^+^, 0.1), 358 (20), 247 (20), 225 (8), 189 (7), 103 (13), 91 (17), 80 (100), 64 (79), 51 (19). Anal. Calcd. for C_24_H_17_ClN_2_O_2_ (400): C, 71.91; H, 4.27; N, 6.99. Found: C, 71.86; H, 4.20; N, 6.91%.

Synthesis of 4-(4-chlorophenyl)-2-oxo-6-(5-oxo-1,3-diphenyl-4,5-dihydro-1*H*-pyrazol-4-yl)-1,2-dihydropyridine-3-carbonitrile **(6)**. A mixture of **5** (0.01 mol), ethylcyanoacetate (0.01 mol) in ethanol (30 mL) containing ammonium acetate (2 gm) was heated under reflux for 24 h. The reaction mixture was allowed to cool and poured onto crushed ice then acidified with HCl. The separated solid was filtered, washed with water and crystallized from ethanol to give pale yellow crystals; yield (84%); m.p. 230–232 °C. IR (KBr, cm^−1^) ν_max_ = 3420 (NH), 3061 (CH-arom), 2926 (CH-aliph), 2208 (CN), 1708 (CO) cm^−1^. ^1^H-NMR (300 MHz, DMSO-*d*_*6*_) δ (ppm): 2.30 (s, 1H, CH-pyrazole), 6.82–8.09 (m, 15H, aromatic H), 9.20 (s, 1H, NH). ^13^C-NMR (100 MHz, DMSO-*d*_*6*_) δ (ppm): 55.1, 101.5, 114.2, 117.8, 123.5, 123.5, 127.2, 127.2, 127.7, 127.7, 127.8, 127.8, 127.8, 127.8, 128.3, 129.1, 129.1, 130.2, 131.6, 131.8, 132.9, 135.2, 136.2, 156.1, 160.5, 164.9, 168.1. MS (EIMS) *m/z*: 466 (M^+^+2, 0.03), 360 (9), 235 (8), 206 (2), 125 (100), 115 (14), 102 (15), 91 (26), 77 (97), 64 (14), 51 (26). Anal. Calcd. for C_27_H_17_ClN_4_O_2_ (464): C, 69.75; H, 3.69; N, 12.05. Found: C, 69.81; H, 3.80; N, 12.11%.

General procedure for the synthesis of hydrazono derivatives **(8a–b)**. To a stirred cold solution of aryldiazonium chlorides **7a–b** (0.01 mol), prepared by treating aniline derivatives (0.01 mol) with sodium nitrite (0.01 mol) in HCl, ethanol (30 mL) and catalytic amount of sodium acetate, the active methyl reagent **2** was added gradually. The stirring was continued for 2 h. The solid product so formed was filtered off, washed with water several times, dried and crystallized from the proper solvent to afford **8a–b**.

4-(2-(2-(4-Chlorophenyl)hydrazono)acetyl)-1,3-diphenyl-1*H*-pyrazol-5(4*H*)-one **(8a)**. It was obtained as an orange crystals from ethanol; yield (95%); m.p. 170–172 °C. IR (KBr, cm^−1^) ν_max_ = 3440 (NH), 3066 (CH-arom), 2927 (CH-aliph), 1772, 1690 (2CO) cm^−1^. ^1^H-NMR (300 MHz, DMSO-*d*_*6*_) δ (ppm): 2.32 (s, 1H, CH-pyrazole), 6.01 (s, 1H, = CH), 6.82–8.14 (m, 14H, aromatic H), 12.00 (s, 1H, NH). MS (EIMS) *m/z*: 418 (M^+^+2, 0.2), 416 (0.2), 374 (38), 263 (15), 235 (18), 129 (26), 99 (19), 77 (100), 64 (19), 51 (23). Anal. Calcd. for C_23_H_17_ClN_4_O_2_ (416): C, 66.27; H, 4.11; N, 13.44. Found: C, 66.32; H, 4.17; N, 13.49%.

4-(2-(2-(4-Methoxyphenyl)hydrazono)acetyl)-1,3-diphenyl-1H-pyrazol-5(4*H*)-one **(8b)**. It was obtained as red crystals from ethanol; yield (92%); m.p. 188–190 °C. IR (KBr, cm^−1^) ν_max_ = 3440 (NH), 3057 (CH-arom), 2928 (CH-aliph), 1720, 1655 (2CO) cm^−1^. ^1^H-NMR (300 MHz, DMSO-*d*_*6*_) δ (ppm): 2.25 (s, 1H, CH-pyrazole), 3.62 (s, 3H, OCH_3_), 6.02 (s, 1H, = CH), 7.25–7.84 (m, 14H, aromatic H), 11.80 (hump, 1H, NH). ^13^C-NMR (100 MHz, DMSO-*d*_*6*_) δ (ppm): 53.5, 54.7, 114.2, 114.2, 115.8, 115.8, 123.7, 123.7, 127, 127.1, 127.1, 127.8, 127.8, 127.8, 127.8, 130.1, 133, 133.7, 134.6, 137.4, 154.7, 155.4, 170.1, 201.6. MS (EIMS) *m/z*: 412 (M^+^, 0.1), 370 (42), 122 (100), 107 (11), 91 (20), 77 (89), 51 (25). Anal. Calcd. for C_24_H_20_N_4_O_3_ (412): C, 69.89; H, 4.89; N, 13.58. Found: C, 69.80; H, 4.86; N, 13.51%.

General procedure for the synthesis of pyrazolopyridazinone derivatives **(9a–b)**. A mixture of **8a–b** (0.01 mol) and ammonium acetate (2.0 gm) was fused for 3.0 min in domestic microwave. The reaction mixture was left to stand, and then triturated with ethanol; the solid product so formed was collected by filtration and crystallized from the proper solvent to give **9a–b**.

7-(4-Chlorophenyl)-1, 3-diphenyl-1*H*-pyrazolo [3,4-c] pyridazin-4(7*H*)-one **(9a)**. It was obtained as an orange crystals from ethanol; yield (95%); m.p. 170–172 °C. IR (KBr, cm^−1^) ν_max_ = 3061 (CH-arom), 1653 (CO) cm^−1^. ^1^H-NMR (400 MHz, DMSO-*d*_*6*_) δ (ppm): 7.26–8.17 (m, 15H, aromatic H and CH-pyridazine). MS (EIMS) *m/z*: 398 (M^+^, 0.01), 354 (74), 353 (8), 325 (2), 263 (9), 235 (14), 167 (5), 129 (21), 91 (45), 77 (100), 51 (20). Anal. Calcd. for C_23_H_15_ClN_4_O (398): C, 69.26; H, 3.79; N, 14.05. Found: C, 69.30; H, 3.86; N, 14.10%.

7-(4-Methoxyphenyl)-1,3-diphenyl-1*H*-pyrazolo[3,4-c]pyridazin-4(7*H*)-one **(9b)**. It was obtained as red crystals from ethanol; yield (92%); m.p. 188–190 °C. IR (KBr, cm^−1^) ν_max_ = 3059 (CH-arom), 2927 (CH-aliph), 1654 (CO) cm^−1^. ^1^H-NMR (400 MHz, DMSO-*d*_*6*_) δ (ppm): 3.81 (s, 3H, OCH_3_), 6.01 (s, 1H, =CH-pyridazine), 6.94–8.19 (m, 14H, aromatic H). ^13^C-NMR (100 MHz, DMSO-*d*_*6*_) δ (ppm): 53.6, 91.8, 114.8, 114.8, 116.2, 116.2, 120.6, 120.6, 124.2, 126.5, 126.5, 127.8, 128.3, 128.3, 128.6, 128.6, 130.7, 137.8, 138.2, 140.0, 142.4, 148.1, 154.0, 166.5. MS (EIMS) *m/z*: 394 (M^+^, 0.1), 338 (2), 236 (40), 207 (5), 167 (2), 128 (21), 115 (10), 103 (53), 91 (57), 77 (100), 64 (91), 51 (16). Anal. Calcd. for C_24_H_18_N_4_O_2_ (394): C, 73.08; H, 4.60; N, 14.20. Found: C, 73.11; H, 4.67; N, 14.20%.

General procedure for the synthesis of 1, 3-diphenyl pyrazolone derivatives **(10a–b)**. A mixture of diphenyl pyrazolone **1** (0.01 mol), appropriate aryl aldehydes **4a–b** (0.01 mol) in ethanol (30 mL) with catalytic amount of piperidine was heated under reflux for 3 h. The reaction mixture was allowed to cool and poured into crushed ice then acidified with HCl. The separated solid was filtered, washed with water and crystallized from an approper solvent to give **10a–b**.

4-(4-Chlorobenzylidene)-1,3-diphenyl-1*H*-pyrazol-5(4*H*)-one **(10a)**. It was obtained as pale yellow crystals from ethanol; yield (80%); m.p. 215–217 °C. IR (KBr, cm^−1^) ν_max_ = 3090 (CH-arom), 1676 (CO) cm^−1^. ^1^H-NMR (300 MHz, DMSO-*d*_*6*_) δ (ppm): 5.14 (s, 1H, CH-oleffinic), 7.11–8.03 (m, 14 H, aromatic H). MS (EIMS) *m/z*: 360 (M^+^+2, 14), 358 (44), 357 (19), 247 (53), 246 (12), 236 (42), 189 (14), 103 (37), 102 (18), 90 (38), 83 (13), 77 (100), 76 (52), 50 (23). Anal. Calcd. for C_22_H_15_N_2_OCl (358): C, 73.64; H, 4.21; N, 7.81. Found: C, 73.69; H, 4.27; N, 7.88%.

4-(4-Hydroxybenzylidene)-1, 3-diphenyl-1*H*-pyrazol-5(4*H*)-one **(10b)**. It was obtained yellow crystals from ethanol; yield (78%); m.p. 212–214 °C. IR (KBr, cm^−1^) ν_max_ = 3448 (OH), 3057 (CH-arom), 1638 (CO) cm^−1^. ^1^H-NMR (300 MHz, DMSO-*d*_*6*_) δ (ppm): 5.09 (s, 1H, CH-oleffinic), 6.58–8.02 (m, 15H, aromatic H and OH). ^13^C-NMR (100 MHz, DMSO-*d*_*6*_) δ (ppm): 114.7, 114.7, 117.2, 117.2, 123.9, 124.3, 127.1, 127.8, 127.8, 127.8, 127.8, 128.7, 128.7, 130.3, 131.5, 131.5, 136.8, 143, 145.1, 154.5, 158.6, 168.2. MS (EIMS) *m/z*: 340 (M^+^, 100), 339 (36), 248 (15), 247 (62), 207 (57), 178 (14), 91 (27), 77 (72), 64 (15), 51 (36). Anal. Calcd. for C_22_H_16_N_2_O_2_ (340): C, 77.63; H, 4.74; N, 8.23. Found: C, 77.65; H, 4.77; N, 8.28%.

General procedure for the Synthesis of pyrazolopyrimidinethione derivatives **(11a–b)**. To boiling solution of compounds **10a–b** (0.01 mol) in ethanolic potassium hydroxide (30 mL, 10%), thiourea (0.01 mol) was added. The reaction mixture was refluxed for 20 h, then allowed to cool and poured into crushed ice then acidified with HCl. The separated solid was filtered, washed with water and crystallized from the proper solvent to give **11a–b**.

4-(4-Chlorophenyl)-1, 3-diphenyl-4, 5-dihydro-1*H*-pyrazolo [3,4-*d*] pyrimidine-6(7*H*)-thione **(11a)**. It was obtained as pale yellow crystals from ethanol/water; yield (76%); m.p. 136–138 °C. IR (KBr, cm^−1^) ν_max_ = 3447, 3400 (2NH), 3057 (CH-arom), 2929 (CH-aliph) cm^−1^. ^1^H-NMR (300 MHz, DMSO-*d*_*6*_) δ (ppm): 6.01 (s, 1H, CH-pyrimidine), 6.98–7.92 (m, 16H, aromatic H + 2NH). MS (EIMS) *m/z*: 418 (M^+^+2, 16), 416 (24), 371 (18), 324 (36), 302 (31), 271 (61), 244 (43), 225 (22), 171 (24), 95 (49), 81 (78), 67 (52), 57 (100), 55 (55). Anal. Calcd. for C_23_H_17_ClN_4_S (416): C, 66.26; H, 4.11; N, 13.44. Found: C, 66.20; H, 4.01; N, 13.37%.

4-(4-Hydroxyphenyl)-1, 3-diphenyl-4, 5-dihydro-1*H*-pyrazolo [3,4-*d*] pyrimidine-6(7*H*)-thione **(11b)**. It was obtained as yellow crystals from ethanol/water; yield (79%); m.p. 137–139 °C. IR (KBr, cm^−1^) ν_max_ = 3576 (OH), 3434, 3400 (2NH), 3055 (CH-arom), 2953 (CH-aliph) cm^−1^. ^1^H-NMR (300 MHz, DMSO-*d*_*6*_) δ (ppm): 6.01 (s, 1H, CH-pyrimidine), 6.69–7.83 (m, 17H, aromatic H, 2NH and OH). ^13^C-NMR (100 MHz, DMSO-*d*_*6*_) δ (ppm): 54.8, 107, 114.6, 114.6, 122.3, 122.3, 127.1, 127.1, 127.8, 128.2, 128.2, 128.2, 129, 129, 130.1, 130.1, 131.2, 134, 140.2, 146.3, 149.3, 157, 180.3. MS (EIMS) *m/z*: 398 (M^+^, 0.2), 236 (74), 194 (10), 149 (6), 123 (10), 103 (58), 91 (56), 77 (100), 69 (82), 57 (84), 51 (31). Anal. Calcd. for C_23_H_18_N_4_OS (398): C, 69.32; H, 4.55; N, 14.06. Found: C, 69.36; H, 4.60; N, 14.12%.

General procedure for the synthesis of pyrazolo[3,4-*d*] thiazolo[3,2-a]pyrimidinone derivatives **(12a–b)**. A mixture of **11a–b** (0.01 mol), chloroacetic acid (0.01 mol) and anhydrous sodium acetate (1.6 g) in acetic acid (30 mL) and acetic anhydride (10 mL) was refluxed for 3 h. The reaction mixture was poured into water. The separated solid was filtered off and crystallized from an approper solvent to give **12a–b**.

4-(4-Chlorophenyl)-1,3-diphenyl-4,7-dihydropyrazolo[3,4-*d*]thiazolo[3,2-a]pyrimidin-6(1*H*)-one **(12a)**. It was obtained as pale yellow crystals from benzene; yield (91%); m.p. 148–150 °C. IR (KBr, cm^−1^) ν_max_ = 3061 (CH-arom), 2928 (CH-aliph), 1709 (CO) cm^−1^. ^1^H-NMR (300 MHz, DMSO-*d*_*6*_) δ (ppm): 5.10 (s, 2H, CH_2_), 5.90 (s, 1H, CH-pyrimidine), 7.10–8.03 (m, 14H, aromatic H). MS (EIMS) *m/z*: 456 (M^+^, 0.3), 236 (13), 194 (3), 125 (4), 91 (33), 77 (100), 63 (78), 51 (25). Anal. Calcd. for C_25_H_17_ClN_4_OS (456): C, 65.71; H, 3.75; N, 12.26. Found: C, 65.75; H, 3.79; N, 12.30%.

4-(4-Hydroxyphenyl)-1,3-diphenyl-4,7-dihydropyrazolo[3,4-*d*]thiazolo[3,2-a]pyrimidin-6(1*H*)-one **(12b)**. It was obtained as brown crystals from benzene; yield (82%); m.p. 152–154 °C. IR (KBr, cm^−1^) ν_max_ = 3438 (OH), 3061 (CH-arom), 2919 (CH-aliph), 1713 (CO) cm^−1^. ^1^H-NMR (300 MHz, DMSO-*d*_*6*_) δ (ppm): 5.10 (s, 2H, CH_2_), 6.00 (s, 1H, CH-pyrimidine), 7.00–8.20 (m, 14H, aromatic H), 9.20 (hump, 1H, OH). ^13^C-NMR (100 MHz, DMSO-*d*_*6*_) δ (ppm): 29.2, 43.8, 114.1, 114.1, 115.4, 121.0, 121.6, 121.6, 125.1, 126.3, 126.3, 127.6, 127.6, 128.1, 128.1, 128.1, 128.3, 128.3, 130.3, 131.8, 138.6, 147.6, 155.3, 160.2, 177.0. MS (EIMS) *m/z*: 438 (M^+^, 0.04), 215 (2), 138 (9), 123 (19), 101 (15), 87 (64), 63 (100), 58 (63), 51 (7). Anal. Calcd. for C_25_H_18_N_4_O_2_S (438): C, 68.48; H, 4.14; N, 12.78. Found: C, 68.41; H, 4.11; N, 12.71%.

Synthesis of 4-(5-oxo-1, 3-diphenyl-1*H*-pyrazol-4-(5*H*)-ylidene)-1,3-diphenyl-1*H*-pyrazol-5-one **(13)**. To a stirred solution of pyrazolone **1** (0.5 gm) in acetic acid (20 mL), sodium nitrite solution (0.02 mol) in water (5 mL) was added dropwise over 10 min. The solid product was collected and recrystallized from ethanol to give orange crystals; yield (88%); m.p. 180–182 °C. IR (KBr, cm^−1^) ν_max_ = 3092 (CH-arom), 1700, 1690 (2CO) cm^−1^. ^1^H-NMR (300 MHz, DMSO-*d*_*6*_) δ (ppm): 7.23–8.06 (m, 20H, aromatic H). ^13^C-NMR (100 MHz, DMSO-*d*_*6*_) δ (ppm): 116.4, 116.4, 116.4, 116.4, 127.2, 127.2, 127.8, 127.8, 127.8, 127.8, 127.8, 127.8, 127.8, 127.8, 128.3, 128.3, 128.3, 128.3, 136.8, 136.8, 130, 130, 141.2, 141.2, 144.9, 144.9, 156.5, 156.5, 167, 167. MS (EIMS) *m/z*: 470 (M^+^+2, 0.07), 265 (61), 220 (25), 167 (5), 129 (29), 115 (14), 91 (29), 77 (100), 51 (32). Anal. Calcd. for C_30_H_20_O_2_N_4_ (468): C, 76.91; H, 4.30; N, 11.96. Found: C, 76.97; H, 4.35; N, 11.99%.

Synthesis of 5-amino-1,3-diphenyl-1*H*-thieno[3,2-c]pyrazole-6-carbohydrazide **(14)**. A mixture of diphenylpyrazolone **1** (0.01 mol), cyanoacetohydrazide (0.01 mol) and sulfur (0.01 mol) in DMF (50 mL) containing catalytic amount of piperidine was heated under reflux for 12 h. The reaction mixture was allowed to cool and poured into crushed ice then acidified with HCl. The separated solid was filtered, washed with water and crystallized from DMF/EtOH to give yellow crystals; yield (78%); m.p. 300–302 °C. IR (KBr, cm^−1^) ν_max_ = 3383, 3292 (2NH_2_), 3169 (NH), 3063 (CH-arom), 1663 (CO) cm^−1^. ^1^H ^1^H-NMR (300 MHz, DMSO-*d*_*6*_) δ (ppm): 6.00 (s, 2H, NH_2_), 7.19–7.91 (m, 12H, aromatic H and NH_2_), 11.20 (hump, 1H, NH). ^13^C-NMR (100 MHz, DMSO-*d*_*6*_) δ (ppm): 104.3, 124.1, 124.1, 125.1, 125.8, 126.5, 126.5, 127.6, 128.6, 128.6, 128.8, 128.8, 131.8, 138.6, 140.9, 163.6, 165.8, 166.8. MS (EIMS) *m/z*: 351 (M^+^+2, 20), 349 (24), 333 (24), 310 (32), 282 (26), 240 (18), 204 (33), 190 (21), 168 (20), 114 (100), 84 (30), 70 (42), 57 (54), 53 (23). Anal. Calcd. for C_18_H_15_N_5_OS (349): C, 61.87; H, 4.33; N, 20.04. Found: C, 61.92; H, 4.37; N, 20.10%.

Synthesis of 5-amino-1,3-diphenyl-1H-thieno[3,2-c]pyrazole-6-(N-ethoxymethylene-carbohydrazide) **(15)**. A mixture of **14** (0.01 mol) and triethylorthoformate (5 mL) in acetic anhydride (10 mL) was heated under reflux for 6 h. The reaction mixture was allowed to cool and poured into crushed ice. The separated solid was filtered, washed with water and crystallized from ethanol to give brown crystals; yield (60%); m.p. 110–112 °C. IR (KBr, cm^−1^) ν_max_ = 3454, 3400 (NH_2_/NH), 3061 (CH-arom), 2979–2852 (CH-aliph), 1661 (CO) cm^−1^. ^1^H-NMR (400 MHz, DMSO-*d*_*6*_) δ (ppm): 1.06 (t, 3H, CH_3_), 4.35 (q, 2H, CH_2_), 7.19–8.36 (m, 13H, aromatic H, =CH and NH_2_), 9.96 (s, 1H, NH). ^13^C-NMR (100 MHz, DMSO-*d*_*6*_) δ (ppm): 17.2, 65.6, 103.8, 123.7, 123.7, 124.4, 126.4, 127.4, 127.4, 127.9, 128.2, 128.2, 129.3, 129.3, 130.8, 140.1, 140.7, 149.4, 165.1, 166.7, 167. MS (EIMS) *m/z*: 405 (M^+^, 5), 236 (52), 215 (20), 103 (44), 90 (36), 89 (12), 77 (100), 64 (30), 50 (27). Anal. Calcd. for C_21_H_19_O_2_N_5_S (405): C, 62.21; H, 4.72; N, 17.27. Found: C, 62.25; H, 4.77; N, 17.31%.

Synthesis of 6-(1,3,4-oxadiazol-2-yl)-1,3-diphenyl-1*H*-thieno[3,2-c]pyrazol-5-amine **(16)**. Compound **15** (0.5 gm) was heated at 120 °C for 30 min. The reaction product was purified preparative TLC on silica gel using chloroform/ethylacetate (80:20) as an eluent to give brown crystals; yield (90%). m.p. 278–280 °C. IR (KBr, cm^−1^) ν_max_ = 3453, 3400 (NH_2_), 3063 (CH-arom) cm^−1^. ^1^H-NMR (300 MHz, DMSO-*d*_*6*_) δ (ppm): 7.20–7.92 (m, 11H, aromatic H and CH-Oxadiazol), 11.34 (s, 2H, NH_2_). ^13^C-NMR (100 MHz, DMSO-*d*_*6*_) δ (ppm): 104.7, 120.3, 123.4, 123.4, 125.7, 126.2, 126.4, 126.4, 127.6, 128.1, 128.1, 128.5, 128.5, 130.8, 140.3, 140.8, 155, 165.8, 167.2. MS (EIMS) *m/z*: 361 (M^+^+2, 36), 310 (28), 270 (42), 252 (35), 233 (34), 193 (34), 158 (43), 134 (32), 123 (37), 91 (36), 80 (100), 63 (46), 51 (31). Anal. Calcd. for C_19_H_13_ON_5_S (359): C, 63.49; H, 3.65; N, 19.49. Found: C, 63.46; H, 3.60; N, 19.43%.

Synthesis of 5-amino-N′-benzoyl-1,3-diphenyl-1*H*-thieno[3,2-c]pyrazole-6-carbohydrazide **(18)**. A solution of **14** (0.01 mol) in acetonitrile (30 mL) was heated under reflux with (0.01 mol) of benzoyl chloride for 7 h. The solid which separated was collected and crystallized from ethanol to give yellow crystals; yield (61%); m.p. 100–102 °C. IR (KBr, cm^−1^) ν_max_ = 3455, 3400, 3161 (NH_2_/NH), 3059 (CH-arom), 1747, 1662 (2CO) cm^−1^. ^1^H-NMR (300 MHz, DMSO-*d*_*6*_) δ (ppm): 6.00 (s, 2H, NH_2_) 7.02–8.12 (m, 17H, aromatic H and 2 NH). MS (EIMS) *m/z*: 455 (M^+^+2, 40), 453 (54), 423 (48), 403 (56), 364 (56), 349 (52), 297 (46), 257 (59), 237 (100), 196 (39), 183 (22), 128 (40), 62 (24). Anal. Calcd. for C_25_H_19_O_2_N_5_S (453): C, 66.21; H, 4.22; N, 15.44. Found: C, 66.25; H, 4.26; N, 15.47%.

Synthesis of N-(8-oxo-1,3-diphenyl-1*H*-pyrazolo [3′,4′:4,5] thieno[2,3-*d*]pyrimidin-7(8*H*)-yl) benzamide **(19)**. A mixture of compounds **18** and (10 mL) of triethyl orthoformate were heated at reflux for 4 h. The reaction mixture was allowed to cool and poured into crushed ice. The separated solid was filtered, washed with water and crystallized from ethanol to give red crystals; yield (67%). m.p. 170–172 °C. IR (KBr, cm^−1^) ν_max_ = 3448 (NH), 3060 (CH-arom), 1700, 1630 (2CO) cm^−1^. ^1^H-NMR (300 MHz, DMSO-*d*_*6*_) δ (ppm): 7.21–8.00 (m, 16H, aromatic H), 9.90 (s, 1H, NH). ^13^C-NMR (100 MHz, DMSO-*d*_*6*_) δ (ppm): 105.3, 121.6, 121.6, 125.1, 126.1, 126.1, 126.2, 126.2, 127.8, 127.8, 127.8, 128.1, 128.3, 128.3, 129.4, 129.4, 130.8, 131.2, 134.1, 138.3, 140.5, 153.6, 159.3, 161.8, 165.3, 166.8. MS (EIMS) *m/z*: 463 (M^+^, 0.2), 405 (11), 320 (71), 290 (34), 274 (27), 262 (35), 246 (37), 103 (48), 91 (39), 77 (100), 57 (28), 51 (16). Anal. Calcd. for C_26_H_17_O_2_N_5_S (463): C, 67.37; H, 3.70; N, 15.11. Found: C, 67.41; H, 3.75; N, 15.16%.

Synthesis of 3-amino-5-(5-amino-1,3-diphenyl-1*H*-thieno[3,2-c]pyrazol-6-yl)-1*H*-pyrazole-4-carbonitrile **(20)**. A mixture of **14** (0.01 mol), malononitrile (0.01 mol) in ethanol (30 mL) containing catalytic amount of piperidine was heated under reflux for 24 h. The reaction mixture was allowed to cool and poured into crushed ice then acidified with HCl. The separated solid was filtered, washed with water and crystallized from dioxane, as brawn crystals; yield (75%); m.p. 280–282 °C. IR (KBr, cm^−1^) ν_max_ = 3450, 3400 (NH_2_/NH), 3060 (CH-arom), 2195 (CN) cm^−1^. ^1^H-NMR (300 MHz, DMSO-*d*_*6*_) δ (ppm): 6.00 (s, 2H, NH_2_), 7.16–7.95 (m, 13H, aromatic H, NH and NH_2_). ^13^C-NMR (100 MHz, DMSO-*d*_*6*_) δ (ppm): 98, 104.8, 113.9, 120.7, 123.4, 123.4, 125.6, 126.1, 126.1, 126.2, 128.1, 128.1, 128.9, 128.9, 128.9, 129.3, 134.2, 140.1, 141.2, 153.8, 167.2. MS (EIMS) *m/z*: 399 (M^+^+2, 2), 397 (3), 236 (27), 194 (6), 103 (25), 91 (42), 79 (100), 64 (69), 56 (44), 51 (31). Anal. Calcd. for C_21_H_15_N_7_S (397): C, 63.46; H, 3.80; N, 24.67. Found: C, 63.50; H, 3.86; N, 24.70%.

Synthesis of (5-amino-1,3-diphenyl-1*H*-thieno[3,2-c]pyrazol-6-yl) (3,5-dimethyl-1*H*-pyrazol-1-yl) methanone **(21)**. A mixture of compound **14** (0.01 mol), and the α,β-diketone (Acetylacetone) (0.01 mol) in absolute ethanol (30 mL) was stirred under reflux for 12 h. The reaction mixture was allowed to cool to 0 °C for 24 h, The separated solid was filtered off, dried and crystallized from dioxane, as brawn crystals; yield (81%); m.p. 270–272 °C. IR (KBr, cm^−1^) ν_max_ = 3439, 3400 (NH_2_), 3060 (CH-arom), 2921–2851 (CH-aliph), 1718 (CO) cm^−1^. ^1^H-NMR (300 MHz, DMSO-*d*_*6*_) δ (ppm): 2.68 (s, 3H, CH_3_), 2.84 (s, 3H, CH_3_), 6.88 (s, 2H, NH_2_), 7.48–7.90 (m, 11H, aromatic H). MS (EIMS) *m/z*: 415 (M^+^+2, 0.07), 413 (0.7), 365 (11), 235 (13), 219 (2), 128 (10), 105 (18), 91 (17), 77 (100), 64 (27), 51 (12). Anal. Calcd. for C_23_H_19_ON_5_S (413): C, 66.81; H, 4.63; N, 16.94. Found: C, 66.84; H, 4.66; N, 16.98%.

General procedure for the synthesis of thieno[3,2-c]pyrazole-6-carbohydrazide derivatives **(22a–b)**. A mixture of compound **14** (0.01 mol), appropriate aryl aldehydes **4a–b** (0.01 mol) in ethanol (30 mL) with catalytic amount of piperidine was heated under reflux for 3 h. The reaction mixture was allowed to cool and poured into crushed ice then acidified with HCl. The separated solid was filtered, washed with water and crystallized from the proper solvent to give **22a–b**.

5-Amino-N′-(4-chlorobenzylidene)-1,3-diphenyl-1*H*-thieno[3,2-c]pyrazole-6-carbohydrazide **(22a)**. It was obtained as pale yellow crystals from ethanol; yield (88%); m.p. 218–220 °C. IR (KBr, cm^−1^) ν_max_ = 3433, 3400 (NH_2_/NH), 3055 (CH-arom), 1630 (CO) cm^−1^. ^1^H-NMR (300 MHz, DMSO-*d*_*6*_) δ (ppm): 5.16 (s, 1H, CH-oleffinic), 7.10–8.04 (m, 17H, aromatic H, NH and NH_2_). MS (EIMS) *m/z*: 473 (M^+^+2, 0.06), 471 (0.09), 358 (27), 247 (32), 236 (27), 103 (25), 91 (32), 77 (100), 64 (14), 51 (31). Anal. Calcd. for C_25_H_18_ON_5_SCl (471): C, 63.62; H, 3.84; N, 14.84. Found: C, 63.68; H, 3.89; N, 14.89%.

5-Amino-N′-(4-hydroxybenzylidene)-1,3-diphenyl-1*H*-thieno[3,2-c]pyrazole-6-carbohydrazide **(22b)**. It was obtained as brawn crystals from ethanol; yield (79%); m.p. 200–202 °C. IR (KBr, cm^−1^) ν_max_ = 3447 (OH), 3423, 3286 (NH_2_/NH), 3056 (CH-arom), 1691 (CO) cm^−1^. ^1^H-NMR (300 MHz, DMSO-*d*_*6*_) δ (ppm): 5.09 (s, 1H, CH-oleffinic), 6.57–8.04 (m, 17H, aromatic H, NH and NH_2_), 9.00 (s, 1H, OH). MS (EIMS) *m/z*: 453 (M^+^, 0.1), 339 (1), 235 (27), 206(1), 103 (22), 91(17), 79 (100), 63 (67), 51 (6). Anal. Calcd. for C_25_H_19_O_2_N_5_S (453): C, 66.21; H, 4.22; N, 15.44. Found: C, 66.24; H, 4.26; N, 15.49%.

General procedure for the Synthesis of thieno[3,2-c]pyrazol-5-yl-acetamide derivatives **(23a–b)**. A solution of compounds **22a–b** (0.01 mol) in acetic anhydride (10 mL) was heated for 15 min. After cooling the solid that was separated was recrystallized from approper solvent to give **23a–b**.

N-(6-(2-(4-chlorobenzylidene)hydrazinecarbonyl)-1,3-diphenyl-1*H*-thieno[3,2-c]pyrazol-5-yl) acetamide **(23a)**. It was obtained as white crystals from benzene; yield (58%); m.p. 134–136 °C. IR (KBr, cm^−1^) ν_max_ = 3440, 3400 (2NH), 3061 (CH-arom), 2950 (CH-aliph), 1681, 1616 (2CO) cm^−1^. ^1^H-NMR (300 MHz, DMSO-*d*_*6*_) δ (ppm): 1.95 (s, 3H, CH_3_), 5.30 (s, 1H, CH-oleffinic), 7.17–8.53 (m, 15H, aromatic H and NH), 10.00 (s, 1H, NH). ^13^C-NMR (100 MHz, DMSO-*d*_*6*_) δ (ppm): 27.2, 104.9, 123.4, 123.4, 124.8, 126.3, 127.1, 127.1, 127.1, 127.2, 127.2, 128, 128.1, 128.1, 128.9, 128.9, 130.6, 131.7, 132.1, 132.1, 138.5, 141.8, 148.3, 165, 167.8, 170.2, 185.5. MS (EIMS) *m/z*: 516 (M^+^+2, 1), 464 (6), 358 (15), 246 (20), 224 (9), 188 (7), 91 (27), 77 (100), 63 (28), 51 (21). Anal. Calcd. for C_27_H_20_ClN_5_O_2_S (514): C, 63.09; H, 3.92; N, 13.63. Found: C, 63.13; H, 3.92; N, 13.63%.

N-(6-(2-(4-hydroxybenzylidene)hydrazinecarbonyl)-1,3-diphenyl-1*H*-thieno[3,2-c]pyrazol-5-yl) acetamide **(23b)**. It was obtained as pale yellow crystals from benzene; yield (68%); m.p. 124–126 °C. IR (KBr, cm^−1^) ν_max_ = 3452, 3400, 3250 (OH, 2NH), 3060 (CH-arom), 2924 (CH-aliph), 1745, 1689 (2CO) cm^−1^. ^1^H-NMR (300 MHz, DMSO-*d*_*6*_) δ (ppm): 1.96 (s, 3H, CH_3_), 5.25 (s, 1H, CH-oleffinic), 7.08–7.98 (m, 14 H, aromatic H), 8.58 (s, 1H, NH), 8.61 (s, 1H, NH), 10.90 (s, 1H, OH). MS (EIMS) *m/z*: 497 (M^+^+2, 2), 495 (8), 451 (36), 398 (20), 353 (26), 307 (31), 244 (34), 206 (73), 167 (26), 125 (28), 93 (64), 81 (98), 70 (40), 55 (100). Anal. Calcd. for C_27_H_21_O_3_N_5_S (495): C, 65.44; H, 4.27; N, 14.13. Found: C, 65.44; H, 4.26; N, 14.13%.

Synthesis of 6-methyl-1,3-diphenyl-1,7-dihydro-8*H*-pyrazolo[3′,4′:4,5]thieno[2,3-d]pyrimidin-8-one **(24)**. A solution of compound **23a–b** (0.01 mol) in an ethanolic sodium ethoxide solution (prepared by dissolving 0.23 g of sodium metal in 30 mL ethanol), was heated under reflux for 12 h. The reaction mixture was evaporated under vacuum to dryness. The separated solid crystallized from benzene to give brawn crystals; yield (68%); m.p. 164–166 °C. IR (KBr, cm^−1^) ν_max_ = 3442 (NH), 3061 (CH-arom), 2922 (CH-aliph), 1712 (CO) cm^−1^. ^1^H-NMR (300 MHz, DMSO-*d*_*6*_) δ (ppm): 1.83 (s, 3H, CH_3_), 7.23–7.52 (m, 11H, aromatic H + NH). ^13^C-NMR (100 MHz, DMSO-*d*_*6*_) δ (ppm): 29.1, 105.3, 123.5, 123.5, 125.3, 126.3, 126.4, 126.4, 127.7, 128.2, 128.2, 129.0, 129.0, 130.6, 140.1, 140.1, 155.3, 156.2, 160.1, 167.2. MS (EIMS) *m/z*: 358 (M^+^, 0.1), 340 (1), 205 (2), 236 (22), 194 (2), 107 (51), 91 (32), 77 (100), 51 (27). Anal. Calcd. for C_20_H_14_N_4_OS (358): C, 67.02; H, 3.94; N, 15.63. Found: C, 67.13; H, 3.93; N, 15.64%.

Preparation of 7-amino-1,3-diphenyl-6-thioxo-1,5,6,7-tetrahydro-8*H*-pyrazolo[3′,4′:4,5] thieno[2,3-*d*]pyrimidin-8-one **(25)**. To a hot ethanolic sodium hydroxide (30 mL), compound **14** (0.01 mol), and carbon disulphide (excess 5 mL) were added. The mixture was heated under reflux for 15 h. The reaction mixture was allowed to cool (0 °C), the separated solid was filtered, washed with water and crystallized from dioxane, as brawn crystals; yield (79%); m.p. 266–268 °C. IR (KBr, cm^−1^) ν_max_ = 3454, 3400 (NH_2_/NH), 3061 (CH-arom), 1712 (CO) cm^−1^. ^1^H-NMR (300 MHz, DMSO-*d*_*6*_) δ (ppm): 7.20–7.92 (m, 11H, aromatic H + NH), 11.31 (s, 2H, NH_2_). ^13^C-NMR (100 MHz, DMSO-*d*_*6*_) δ (ppm): 105.3, 125.1, 125.1, 126.1, 127.1, 127.1, 127.1, 127.1, 128.3, 128.3, 128.9, 128.9, 130.6, 138.7, 140.8, 161.3, 168.1, 169.2, 185.6. MS (EIMS) *m/z*: 393 (M^+^+2, 5), 391 (65), 323 (84), 279 (58), 253 (91), 200 (67), 178 (100), 112 (65), 90 (61), 51 (58). Anal. Calcd. for C_19_H_13_N_5_S_2_O (391): C, 58.29; H, 3.35; N, 17.89. Found: C, 58.32; H, 3.36; N, 17.91%.

## Conclusions

The research study reports the successful synthesis and antimicrobial activity of new pyrazolone, pyrazolopyridazine, pyranopyrazole, pyrazolopyrimidine, pyrazolothiazolopyrimidinone, thiazolopyrimidine, thienopyrazole and pyrazolothienopyrimidine derivatives. The antimicrobial study revealed that all the tested compounds showed moderate to good antimicrobial and antifungal activities against pathogenic strains.
