# Correction to: Synthesis, characterization and in vitro antimicrobial activity of novel fused pyrazolo[3,4-*c*]pyridazine, pyrazolo[3,4-*d*]pyrimidine, thieno[3,2-*c*] pyrazole and pyrazolo[3′,4′:4,5]thieno[2,3-*d*]pyrimidine derivatives

**DOI:** 10.1186/s13065-017-0351-8

**Published:** 2017-11-30

**Authors:** Mohamed A. M. Abdel Reheim, Safaa M. Baker

**Affiliations:** Department of Chemistry, Faculty of Science, Arish University, Arish, 45511 Egypt

## Correction to: Chemistry Central Journal (2017) 11:112 10.1186/s13065-017-0339-4

After the publication of this work [1] it was noticed that on page 3 of the article beginning in the paragraph—‘Hydrazide 14 is used as a key producer…’ the compound—“Not ethyl-*N*′-5-amino-1,3-diphenyl-1*H*-thieno[3,2-*c*]pyrazole-6-carbonylformohydrazonate” should be deleted. Also on page 10 of the PDF beginning in paragraph—‘Synthesis of 5-amino-…’ the compound—“Not ethyl-*N*′-5-amino-1,3-diphenyl-1*H*-thieno[3,2-*c*]pyrazole-6-carbonylformohydrazonate” should be deleted. We apologise for these errors.

The original article was corrected.
